# Plastid control of abaxial-adaxial patterning

**DOI:** 10.1038/srep15975

**Published:** 2015-11-02

**Authors:** Eduardo Mateo-Bonmatí, Rubén Casanova-Sáez, Víctor Quesada, Andrea Hricová, Héctor Candela, José Luis Micol

**Affiliations:** 1 Instituto de Bioingeniería, Universidad Miguel Hernández, Campus de Elche, 03202 Elche, Spain

## Abstract

Translational regulation, exerted by the cytosolic ribosome, has been shown to participate in the establishment of abaxial-adaxial polarity in *Arabidopsis thaliana*: many hypomorphic and null alleles of genes encoding proteins of the cytosolic ribosome enhance the leaf polarity defects of *asymmetric leaves1* (*as1*) and *as2* mutants. Here, we report the identification of the *SCABRA1* (*SCA1*) nuclear gene, whose loss-of-function mutations also enhance the polarity defects of the *as2* mutants. In striking contrast to other previously known enhancers of the phenotypes caused by the *as1* and *as2* mutations, we found that *SCA1* encodes a plastid-type ribosomal protein that functions as a structural component of the 70S plastid ribosome and, therefore, its role in abaxial-adaxial patterning was not expected.

The abaxial-adaxial patterning of plant lateral organs, such as the leaves, is known to depend on a complex regulatory network that involves microRNAs, trans-acting siRNAs and several families of transcription factors^1^. Translational regulation, exerted by the cytosolic ribosome, has also been shown to participate in the establishment of abaxial-adaxial polarity, although its role is much less well understood. In the model plant *Arabidopsis thaliana* (hereafter, Arabidopsis), loss-of-function mutations in many different subunits of the cytosolic ribosome specifically cause a dose-dependent syndrome, with phenotypes ranging from embryonic and gametophytic lethality to mild defects in organ growth and polarity. Many such mutations also enhance the leaf polarity defects of *asymmetric leaves1* (*as1*) and *as2* mutants^2^,^3^,^4^,^5^. One mechanism explaining how the cytosolic ribosome influences leaf polarity depends on the presence of uORFs (upstream open reading frames) in the transcripts of several ARF (auxin response factors) involved in abaxial-adaxial patterning^6^.

Considerable efforts have been devoted to the elucidation of the function of nucleus-encoded Plastid Ribosomal Proteins (PRPs) in Arabidopsis, most often using publicly available T-DNA insertion mutants. However, 16 out of 36 of the Arabidopsis nuclear-encoded PRPs are essential proteins, precluding ascertaining their role in post-embryonic tissues^7^.

Here we describe the identification of the Arabidopsis *SCABRA1* (*SCA1*) gene, whose partial loss-of-function enhances the polarity defects caused by *as2* alleles. However, contrary to other previously known *as1* and *as2* enhancers, we found that *SCA1* encodes a plastid-type ribosomal protein that functions as a structural component of the 70S plastid ribosome and, therefore, its role in abaxial-adaxial patterning was unexpected. *SCA1* was already annotated as *EMBRYO DEFECTIVE 3113* (*EMB3113*), an embryonic lethal gene; the *sca1-1* viable allele that we isolated has allowed us studying the role of *SCA1* on leaf morphogenesis.

## Results and Discussion

**Figure 1:**
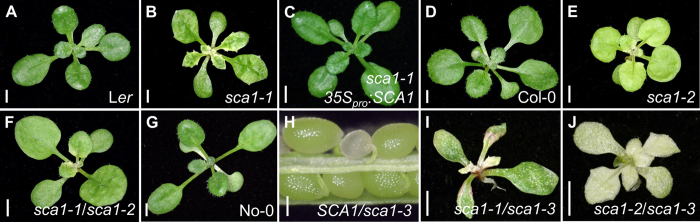
Mutations in the *SCA1* gene alter leaf morphology and pigmentation. (**A–G,I,J**) Rosettes from the (**A**) *Ler*, (**D**) Col-0, and (**G**) No-0 wild types, the (**B**) *sca1-1*, (E) *sca1-2*, (**F**) *sca1-1/sca1-2*, (**I**) *sca1-1/sca1-3*, and (**J**) *sca1-2/sca1-3* mutants, and (**C**) the *sca1-1 35S*_*pro*_*:SCA1* transgenic line. (**H**) Dissected silique from a *SCA1/sca1-3* plant showing a *sca1-3/sca1-3* aborted seed. Unless otherwise stated, all plants are homozygous for the mutations shown. Pictures were taken (**A–G,I,J**) 16 and (**H**) 40 days after stratification (das). Scale bars indicate (**A-G, I, J**) 2  mm, and (**H**) 0.2  mm.

The *scabra1-1* (*sca1-1*) mutant was isolated in a large-scale screen for ethyl methanesulfonate (EMS)-induced mutants with abnormal leaf morphology^8^. The *sca1-1* mutant was assigned to the Scabra phenotypic class, which comprises six additional recessive mutants, all with pale green leaves, uneven leaf surface and prominent marginal teeth. The *sca1-1* mutant exhibits a general reduction in size, which translates into rosettes with significantly reduced projected area when compared with its wild type, Landsberg *erecta* (L*er*) (Fig. 1A,B; Supplementary Figure S1A).

**Figure 2:**
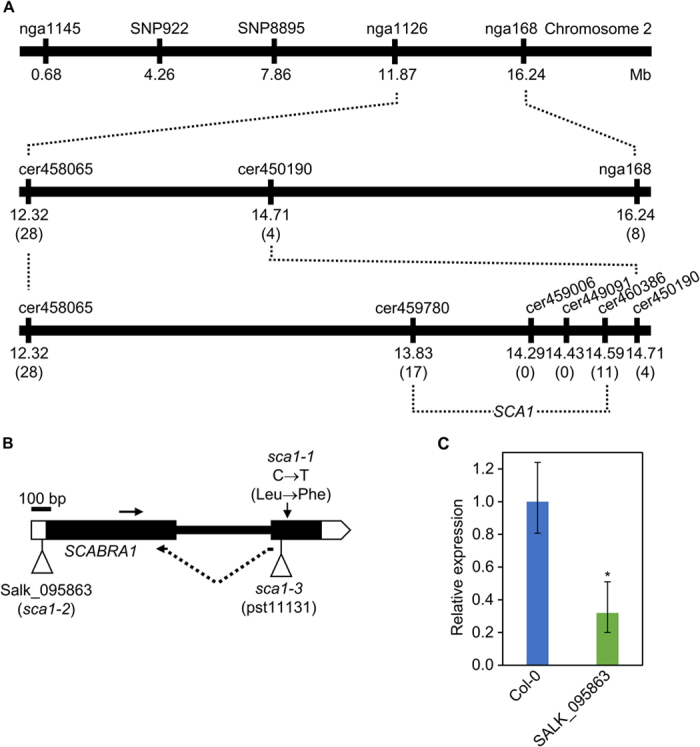
Fine-mapping of the *sca1-1* mutation, structure of the *SCA1* gene, and molecular characterization of the *sca1-2* allele. (**A**) Linkage analysis of the *sca1-1* mutation. The names and physical map positions of the molecular markers used for linkage analysis are shown. All values not in parentheses indicate Mb. The number of recombinant chromosomes found are indicated in parentheses. (**B**) Structure of the *SCA1* gene with indication of the position and nature of *sca1* mutations. Horizontal arrows indicate the oligonucleotides (not drawn to scale) used as primers for determining the relative expression of *SCA1*. The vertical arrow marks the position of the point mutation in *sca1-1*. Triangles represent a T-DNA insertion in *sca1-2* and a *Ds* transposon insertion in *sca1-3*. (**C**) qRT-PCR relative expression analysis of *SCA1* in the SALK_095863 (*sca1-2*) line background. Bars indicate relative expression levels, determined by the comparative C_T_ method, and normalized with the expression of the 18S rRNA housekeeping gene. Error bars indicate the interval delimited by 2^–(ΔΔCT±SD)^. Asterisks indicate ΔC_T_ values significantly different from those of Col-0 in a Mann–Whitney U-test (*p* < 0.01; *n* = 9).

To identify the *SCA1* gene, we followed a strategy combining map-based cloning and next-generation sequencing. We first mapped the *sca1-1* mutation to a 760-kb candidate interval on chromosome 2 using 910 chromosomes (Fig. 2A). We next sequenced the *sca1-1* genome using the Illumina HiSeq2000 platform. After discarding all the putative L*er*/Col-0 polymorphisms, we identified five nucleotide substitutions of the type induced by EMS (three G→A and two C→T transition mutations) within the candidate interval (Supplementary Table 1; see Methods). The C→T mutation in the At2g33800 gene was predicted to cause a Leu→Phe amino acid substitution at residue 233 of the protein. This substitution was confirmed using conventional Sanger sequencing in *sca1-1* and L*er* plants. Additional evidence that At2g33800 is the same gene as *SCA1* was obtained using a construct carrying the coding sequence of At2g33800 placed downstream of the 35S promoter (*35S*_*pro*_*:SCA1*). This construct fully complemented the mutant phenotype of *sca1-1* plants, demonstrating that their phenotype is a consequence of reduced At2g33800 function (Fig. 1A–C; Supplementary Figure S1A).

At2g33800 encodes Plastid Ribosomal Protein S5 (PRPS5), a structural component of the plastid ribosome^7^. We studied two additional lines, which carry insertions in At2g33800: SALK_095863, with a T-DNA insertion in the 5′ untranslated region, and pst11131, with a *Ds* element inserted in the second exon (Fig. 2B). Plants homozygous for the SALK_095863 insertion showed a compact and small rosette with pale-green, roundish leaves and a general size reduction (Fig. 1D,E; Supplementary Figure S1B). The F_1_ progeny of *sca1-1* x SALK_095863 crosses displayed a mutant phenotype, showing that both mutations are allelic (Fig. 1F). Using real-time quantitative RT-PCR (qRT-PCR), we showed that the levels of At2g33800 transcripts were reduced in SALK_095863 to ~40% of the wild-type levels (Fig. 2B,C), suggesting that the insertion behaves as a hypomorphic allele. No homozygotes were found for the pst11131 allele, but we observed 25% aborted seeds in the siliques of hemizygous plants, showing that this allele is embryo-lethal (Fig. 1G,H). Indeed, At2g33800 is also known as *EMBRYO DEFECTIVE 3113* (*EMB3113*)^9^. The F_1_ progeny of crosses involving *sca1-1* and hemizygous pst11131 plants also displayed defects more severe than those of *sca1-1* (Fig. 1B,I). We named *sca1-2* and *sca1-3* the alleles present in the SALK_095863 and pst11131 lines, respectively. The *sca1-2/sca1-3* heterozygotes were viable and exhibited stronger defects than the *sca1-2* homozygotes (Fig. 1E,J).

**Figure 3:**
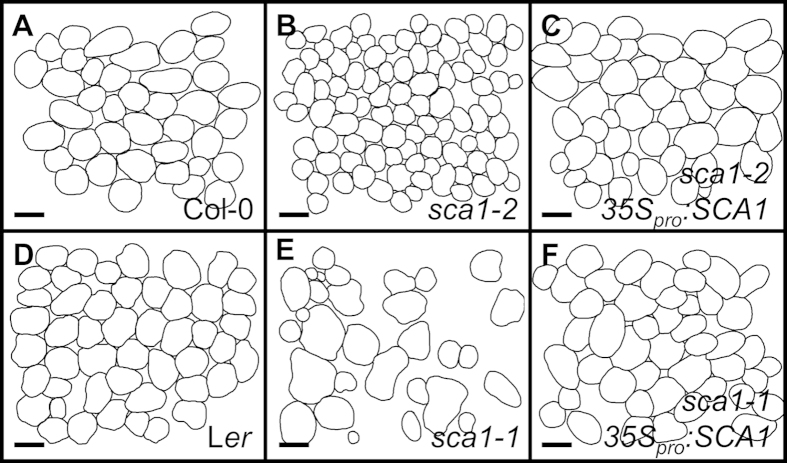
Representative diagrams of the sub-epidermal layer of palisade mesophyll cells from a first leaf of the (A) Col-0 and (D) *Ler* wild types, the (B) *sca1-2*, and (E) *sca1-1* mutants, and the *35S*_*pro*_*:SCA1* transgenic line in the (C) *sca1-2* and (F) *sca1-1* genetic backgrounds, respectively. Diagrams were drawn from differential interference contrast images taken from cleared leaves. Scale bars indicate 50 μm.

The viability of *sca1-1* and *sca1-2* mutants offers an opportunity to study the function of PRPS5 in post-embryonic tissues. Because *sca1* leaves are pale green, we determined their levels of chlorophyll and carotenoids: both mutants had significantly lower levels of chlorophyll *a* and chlorophyll *b*. This reduction was more pronounced in *sca1-2* than in *sca1-1.* The *sca1-2* mutant also had reduced carotenoid levels (Supplementary Figure S2A). Consistent with these results, the maximum efficiencies of photosystem II, measured as *F*_*v*_/*F*_*m*_, were significantly reduced in *sca1* mutants compared with their wild types (Supplementary Figure S2B). In addition to the defects in chloroplast function, mesophyll development was severely perturbed. In paradermal sections, s*ca1-2* exhibited significantly smaller palisade mesophyll cells (Fig. 3A,B; Supplementary Figure S3A). A more severe defect was observed in the mesophyll of *sca1-1* (Fig. 3D,E), which had large intercellular spaces and a distribution of cell sizes wider than in the wild type (Supplementary Figure S3B). The mesophyll phenotype was fully complemented by the *35S*_*pro*_*:SCA1* transgene (Fig. 3C,F; Supplementary Figure S3A).

**Figure 4:**
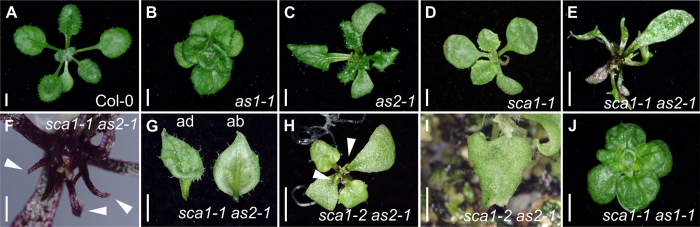
Genetic interaction between the *SCA1* and *AS* genes. Rosettes from (**A**) Col-0, (**B**) *as1-1*, (**C**) *as2-1*, (**D**) *sca1-1* introgressed in Col-0 mutants, and (**E**) *sca1-1 as2-1*, (**H**) *sca1-2 as2-1* and (**J**) *sca1-1 as1-1* double mutants. (**G,I**) Peltate leaves from the (**G**) *sca1-1 as2-1* double mutant, showing the adaxial (ad) and abaxial (ab) sides of the leaf, and (**I**) *sca1-2 as2-1*. (**F**) Detail of the radialized leaves of the *sca1-1 as2-1* double mutant. Pictures were taken (**A**–**F,H,J**) 16, (**G**) 30 and (**I**) 24 das. Scale bars indicate (**A–E,G,J**) 2 mm and (**F**) 0.5 mm.

**Figure 5:**
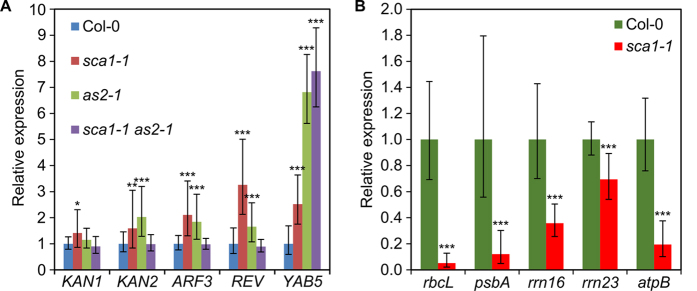
Relative expression analysis of (A) the abaxial-adaxial polarity genes *KANADI1 (KAN1), KAN2, AUXIN RESISTANT FACTOR3 (ARF3), REVOLUTA (REV)* and *YABBY5 (YAB5)* in Col-0, s*ca1-1*, *as2-1* and *sca1-1* *as2-1* plants collected 10 das, and of (B) the plastid-encoded genes *rbcL*, *psbA, rrn16, rrn23* and *atpB*, in Col-0 and *sca1-1* plants collected 10 das. Bars indicate relative expression levels. Error bars indicate the interval delimited by 2^–(ΔΔCT±SD)^. Asterisks indicate ΔC_T_ values significantly different from those of Col-0 in a Mann–Whitney U-test (*p < 0.05, **p < 0.01, ***p < 0.001; n = 9).

Previous authors have reported an enhancement of the phenotype of *as1* mutants in some chloroplast-defective backgrounds, including the *sca3* mutant found in our screen^10^. *SCA3* encodes the plastid-targeted RpoTp RNA polymerase, which is required for the expression of plastid-encoded transcripts^11^. To investigate whether defects in plastid ribosomal proteins can also enhance the abaxial-adaxial polarity defects of *as1* and *as2* mutants (Fig. 4A–C), we isolated *sca1 as1* and *sca1 as2* double mutants. Both *sca1-1* and *sca1-2* enhanced the phenotype of *as2-1* though to a different extent (Fig. 4E,H). A strong abaxialization was observed in *sca1-1 as2-1* plants, which displayed *as2*-like cotyledons, radial leaves (Fig. 4E,F) and some trumpet-shaped leaves (Fig. 4G), an enhancement that occurred with full penetrance and similar expressivity in all double mutant plants. A milder enhancement was seen in *sca1-2 as2-1* plants, which only occasionally had trumpet-shaped leaves (Fig. 4H,I), as expected if the *sca1-2* allele is weaker than *sca1-1*, as suggested by our morphologic analysis. The same enhancement was not apparent in the s*ca1-1 as1-1* double mutant (Fig. 4J). To investigate the molecular basis of this interaction, we examined the expression of abaxial-adaxial polarity markers in *sca1-1 as2-1* plants, including members of the *KANADI* (*KAN*)*, AUXIN RESPONSE FACTOR* (*ARF*)*, YABBY* (*YAB*) and HD-ZIPIII families of transcription factors, using qRT-PCR (Fig. 5A). Compared with Col-0, all the studied genes were upregulated in *sca1-1* and *as2-1*, except for *KAN1*, which was upregulated only in *sca1-1*, specially the HD-ZIPIII gene *REVOLUTA* (*REV*) in *sca1-1* and *YAB5* in both mutants. On the contrary, in *sca1-1 as2-1* plants, the *KAN1*, *KAN2*, *ARF3* and *REV* genes exhibited transcript levels similar to those of the wild type, except for *YAB5*, which was upregulated.

We hypothesized that a dual role of SCA1 as a component of both the cytosolic and plastid ribosomes might explain the observed enhancement. However, this possibility is precluded by the predicted plastid localization signal in the SCA1 protein, the absence of SCA1 from the 80S ribosome in proteomic studies, and the very low similarity between the amino acid sequences of SCA1 and its counterpart in the cytosolic ribosome. In line with a role of SCA1 acting specifically in the plastids, we instead found that the *sca1-1* mutation causes a general change in the expression of the plastid-encoded *rbcL*, *psbA*, *rrn16*, *rrn23* and *atpB* genes, all of which were found to be downregulated in the mutant using qRT-PCR (Fig. 5B). Remarkably, mutations in components of the plastid transcriptional machinery (e.g. *SCA3*) and the plastid translational machinery [e.g. *EMBRYO DEFECTIVE DEVELOPMENT1* (*EDD1*)] have been reported to cause similar defects in leaf abaxial-adaxial polarity, uncovering a role of plastids in the establishment of adaxial fate beyond other known roles in leaf morphogenesis^10^. The inhibition of plastid translation by lincomycin also altered abaxial-adaxial patterning, a defect that was dependent on the *GENOME UNCOUPLED1* gene, which is involved in chloroplast retrograde signaling^12^.

The leaves are lateral organs highly specialized in light capture and photosynthesis, as reflected by their planar shape and the functional differentiation that is apparent along the abaxial-adaxial axis: the tight packing of the adaxial palisade mesophyll provides a solution to maximize light capture, while the abundant stomata and the spongy mesophyll on the abaxial side facilitate gas exchange. Considering that abaxial-adaxial polarity might have evolved as an innovation of land plants to optimize photosynthesis, a functional relationship between plastids and pattern formation is not fully unexpected. On the one hand, chloroplast biogenesis can be seen as a step towards the differentiation of the photosynthetically active palisade mesophyll. On the other, normal chloroplast activity might represent a checkpoint that controls the progression towards the acquisition of adaxial fate. Further study of mutants impairing chloroplast function, such as the ones described in this report, should help to understand how defective chloroplast development feeds back on the establishment of adaxial fate in plant leaves.

## Materials and Methods

### Plant material and growth conditions

*Arabidopsis thaliana* (L.) Heynh. wild-type accessions No-0, Col-0 and L*er*, and the T-DNA insertional line *sca1-2* [SALK_095863 (N595863)] were obtained from the Nottingham Arabidopsis Stock Centre (NASC). The transposon tag line *sca1-3* (pst11131) was obtained from the RIKEN collection. The *sca1-1* mutant was isolated after EMS mutagenesis of L*er* seeds as previously described by Berná *et al.*^8^. All plants were grown on half-strength Murashige and Skoog (MS) agar medium (2.15 g l^−1^ [Duchefa], pH 5.7, and 1% sucrose), at 20 ± 1 °C and 60–70% relative humidity under continuous fluorescent light (~75 μmol m^−2^ s^−1^) as previously described by Ponce *et al.*^13^. Crosses and allelism tests were performed as described by Berná *et al.*^8^. To analyze the genetic interactions of *sca1-1* with *as1-1* and *as2-1,* we standardized the genetic backgrounds, outcrossing *sca1-1* three times to Col-0.

### Cloning-by-sequencing

In order to clone the *SCA1* gene, we used the approach described in Mateo-Bonmatí *et al.*^14^. In brief, we first defined a broad candidate interval of 760 kb (Fig. 2A) by linkage analysis as described in Ponce *et al.*^15^, using the primers listed on Supplementary Table 2, and then we re-sequenced the *sca1-1* genome. After filtering all the putative L*er*/Col-0 polymorphisms, a short list of EMS-type substitutions was obtained (Supplementary Table 1). A total of 3 G → A and 2 C → T substitutions were found within the candidate interval, of which only the C → T substitution in At2g33800 changed the protein sequence. DNA samples were sequenced by Fasteris (Geneva, Switzerland) using the Illumina HiSeq2000 platform. Paired-end reads were 100-bp long. A total of 89,797,616 reads were obtained, which correspond to a 50.38x sequencing depth. The raw data have been deposited at the Short Read Archive database (http://www.ncbi.nlm.nih.gov/sra) with accession number SRP050297.

### Gene constructs and plant transformation

The *35S*_*pro*_*:SCA1* transgene was made amplifying the full-length coding sequence of At2g33800 from Col-0 cDNA using Phusion polymerase (Thermo Scientific) and primers containing *attB1* and *attB2* sites (Supplementary Table 2). The amplification product was cloned into the pGEM-T Easy221 donor vector (kindly provided by Prof. B. Scheres) using BP Clonase II (Life Technologies). The insert was sequenced using an ABI PRISM 3130xl Genetic Analyser (Applied Biosystems), and then transferred into the pMDC32 destination vector^16^ using LR Clonase II (Life technologies). The construct was then mobilized into *Agrobacterium tumefaciens* GV3101 (C58C1 Rif^R^) electrocompetent cells, which were used to transform Arabidopsis plants by the floral dip method^17^. T_1_ transformants were selected on agar medium supplemented with 15 μg ml^−1^ hygromycin B (Invitrogen).

### RNA isolation, cDNA synthesis and qRT-PCR

Total RNA was extracted using TRI Reagent (Sigma) from a single Col-0 seedling collected 21 days after stratification (das) to synthesize the cDNA used for preparing the *35S*_*pro*_*:SCA1* transgene and a pool of 10 seedlings (collected 10 das) for quantitative RT-PCR analysis, respectively. DNA was removed using the TURBO DNA-free Kit (Invitrogen). First-strand cDNA was synthesized using random hexamers and the Maxima Reverse Transcriptase system (Fermentas). The 18S rRNA^18^ and *ACTIN2*^10^ genes were used as internal controls in the relative expression analyses of plastid-encoded and dorsoventrality genes, respectively. Three different biological replicates and triplicate reactions were used. PCR mixes were prepared in a volume of 20 μl by adding 7.5 μl of Maxima SYBR Green/ROX qPCR Master Mix (Fermentas), 5 μl of the corresponding primer pair (1.5 μM each), and 1 μl of cDNA template. Relative quantification of gene expression data was performed using the comparative C_T_ method^19^ on a Step One Plus System (Applied Biosystems). The primer sets used are listed on Supplemental Table 2.

### Morphometry

Rosette area measurements and the morphometric analysis of palisade cells of leaves were performed as described previously^20^,^21^. In brief, ten first-node leaves were manually excised and immediately kept in 70% ethanol. Samples were then incubated in a clearing solution (80 g chloral hydrate in 30 ml water) until photosynthetic tissues became transparent and veins were visible. Whole leaves were mounted on glass slides in solutions of 80 g chloral hydrate, 20 ml glycerol and 10 ml water. Pictures from palisade mesophyll were taken halfway along the primary vein and the leaf margin.

### Pigment determination and photosynthesis analysis

For determination of chlorophylls and carotenoids, five independent samples of 100 mg each of fresh first-node and second-node leaves from rosettes collected 14 das were pooled, frozen in liquid N_2_, and homogenized with 4 ml of 80% acetone at 4 °C. The samples were centrifuged for 5 min at 2350 g and the pigment concentration in the supernatant was spectrophotometrically determined as previously described^22^. Photosynthetic maximum quantum yield was measured 20 das on plants dark-adapted for 30 min and after applying a 0.8-sec saturating light pulse (4000 μmol m^−2^ sec^−1^). Measurements were made with a DUAL-PAM/F fluorometer and a DUAL-BA leaf-positioning device (WALZ).

## Additional Information

**How to cite this article**: Mateo-Bonmatí, E. *et al.* Plastid control of abaxial-adaxial patterning. *Sci. Rep.* **5**, 15975; doi: 10.1038/srep15975 (2015).

## Supplementary Material

Supplemental Data
