# Supplementary material for: Plastid control of abaxial-adaxial patterning
Source: Sci Rep. 2015 Nov 2;5:15975. doi: 10.1038/srep15975 (PMC4629159; doi:10.1038/srep15975)
Supplement: Supplemental Data [file srep15975-s1.pdf]

Plastid control of abaxial-adaxial patterning

Eduardo Mateo-Bonmatí, Rubén Casanova-Sáez, Víctor Quesada, Andrea Hricová, Héctor Candela, and José Luis Micol

Instituto de Bioingeniería, Universidad Miguel Hernández, Campus de Elche, 03202 Elche, Spain

SUPPLEMENTARY DATA

Supplementary Table S1. Mutations found in the *sca1-1* candidate interval.

Mutation	Region affected	Predicted effect
G→A	At2g32990, 3rd exon	Glu265→Glu (Synonymous)
G→A	At2g33060, 3'UTR	-
C→T	At2g33170, 2nd exon	Ile1011→Ile (Synonymous)
C→T	At2g33800, 2nd exon	Leu233→Phe
G→A	At2g34670, 5'UTR	-

Supplementary Table S2. Primer sets used in this work.

Purpose	Oligonucleotide names	Oligonucleotide sequence (5' → 3')	
		Forward primer (F)	Reverse primer (R)
Linkage analysis	cer458065_F/R	CTGATCTTTTCAACATACATTATAG	GTTATTATTTCCATACTTTTTGGC
	cer450190_F/R	GGAGGGTTAGTTTATAATTG	GGTCGTTATTGTTAAATTATTG
	cer460386_F/R	TGGGTGCCACATAAAAAGGT	TCCATCAACGTTATTTTTCTCAAA
	cer449091_F/R	CCTCTTTCGTATAATTTGTG	CATTACACTACATATTCTGAATAG
	cer459006_F/R	TGGTATGCTCATGTATCGCTTA	AGAGCTGATGAACAAAAGACTGA
	cer459780_F/R	CCAAAACAACAAAGGATTAGTGGA	CCTTGCCTGAAAGAAAAGTGAA
SCA1 sequencing	At2g33800_F/R	TGTTGCTTCCAAGCTGTTTCC	AAGTATTCTACTTTCCCTCACAG
	At2g33800_R2		CACAATCAATTAGAAGAATCGGTT
Gateway cloning	SCA1cds_F/R	GGGGACAAGTTTGTACAAAAAAGCAGGCT	GGGGACCACTTTGTACAAGAAAGCTGG
		ATGGCGACAGCATCAGCTCT	GTATCATCCAAGTAGTAGTATGAGC
Genotyping	Salk_095863_LP/RP	AGCAGATTTCTGAACAGCAGC	AATTAACGTTGCTCGTTGGTG
	LbB1.3_R		ATTTTGCCGATTTCGGAAC
	pst_11131_LP/RP	GGATGTGATGATTTGCTTGG	GGCACAAGTCTTCGCATGTA
	Ds5-1a_R		ACGGGATCCCGGTGAAACGGT
qRT-PCR	18SrRNA_qPCR_F/R	AGGATCCATTGGAGGGCAAGT	CGACGTTTTTAACTGCAACAACT
	qSCA1_F/R	TCAGGTATTGGTGGGAAGCC	CCCTCTGATCTGTGAGGGAAAG
	qACTIN2_F/R	GCACCCTGTTCTTCTTACCG	AACCCTCGTAGATTGGCACA
	rbcL_F/R	GTGTTGGGTTCAAAGCTGGT	CATCGGTCCACACAGTTGTC
	psbA_F/R	GAGCAGCAATGAATGCGATA	CCTATGGGGTCGCTTCTGTA
	rrn16_F/R	CGGTATCTGGGGAATAAGCA	GATTTGACGGCGGACTTAAA
	rrn23_F/R	GGGCGACTGTTTACCAAAAA	TTACCCGACAAGGAATTTCG
	atpB_F/R	GAGCTCGTATGAGAGTTGGT	ACCCAATAAGGCGGATACCT
	qKAN1_F/R	GATCCAGCATTCAAAATCAGG	TTTCTCGTGCCAATCTGGTCT
	qKAN2_F/R	TTTGCATGGGAAGTTAATCG	TTGTTCCCGAGATGCTTGAT
	qARF3_F/R	GGAAAGCCTGATATCCCTGTC	ACCATCCGAACAAGTGTTGA
	qREV_F/R	ATATTCGATGAATCGGGTCGTA	ATAACTCACATGTCTTCCCATCG

This list of oligonucleotides does not include those used for low-resolution mapping, which are described in Ponce *et al.*[15].

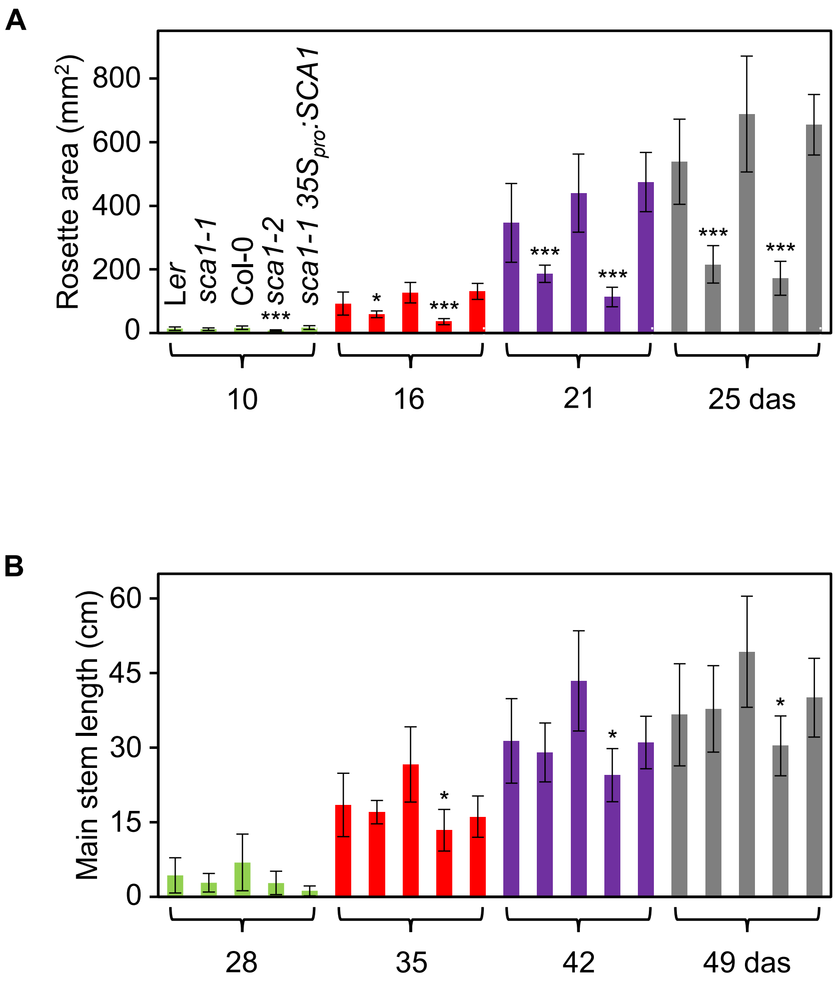

Supplementary Figure S1. Characterization of the growth of the *sca1* mutants. (A) Rosette area progression. (B) Main stem length progression. Asterisks indicate a significant difference with the corresponding wild type in a (A) Student's *t* test (n=16) or (B) Mann-Whitney test (n=8) (**p*<0.05,****p*<0.001).

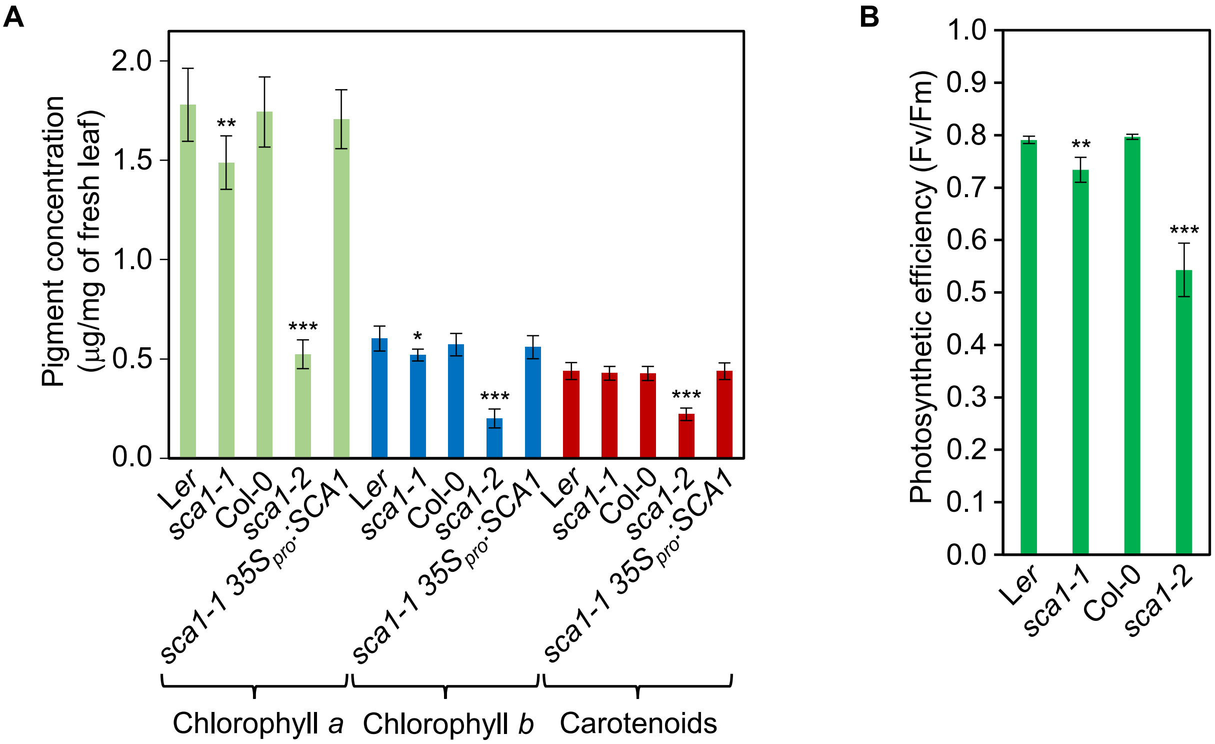

Supplementary Figure S2. Chlorophyll and carotenoid content and photosyntetic efficiency in the *sca1* mutants. (A) Chlorophyll and carotenoid concentration in plant leaves collected 14 das. (B) Maximum efficiencies of PSII measured as *Fv/Fm* in plant leaves at 20 das. Asterisks indicate a significant difference with the corresponding wild type in a Mann-Whitney test (n=8) (*p<0.05,**p<0.01,***p<0.001).

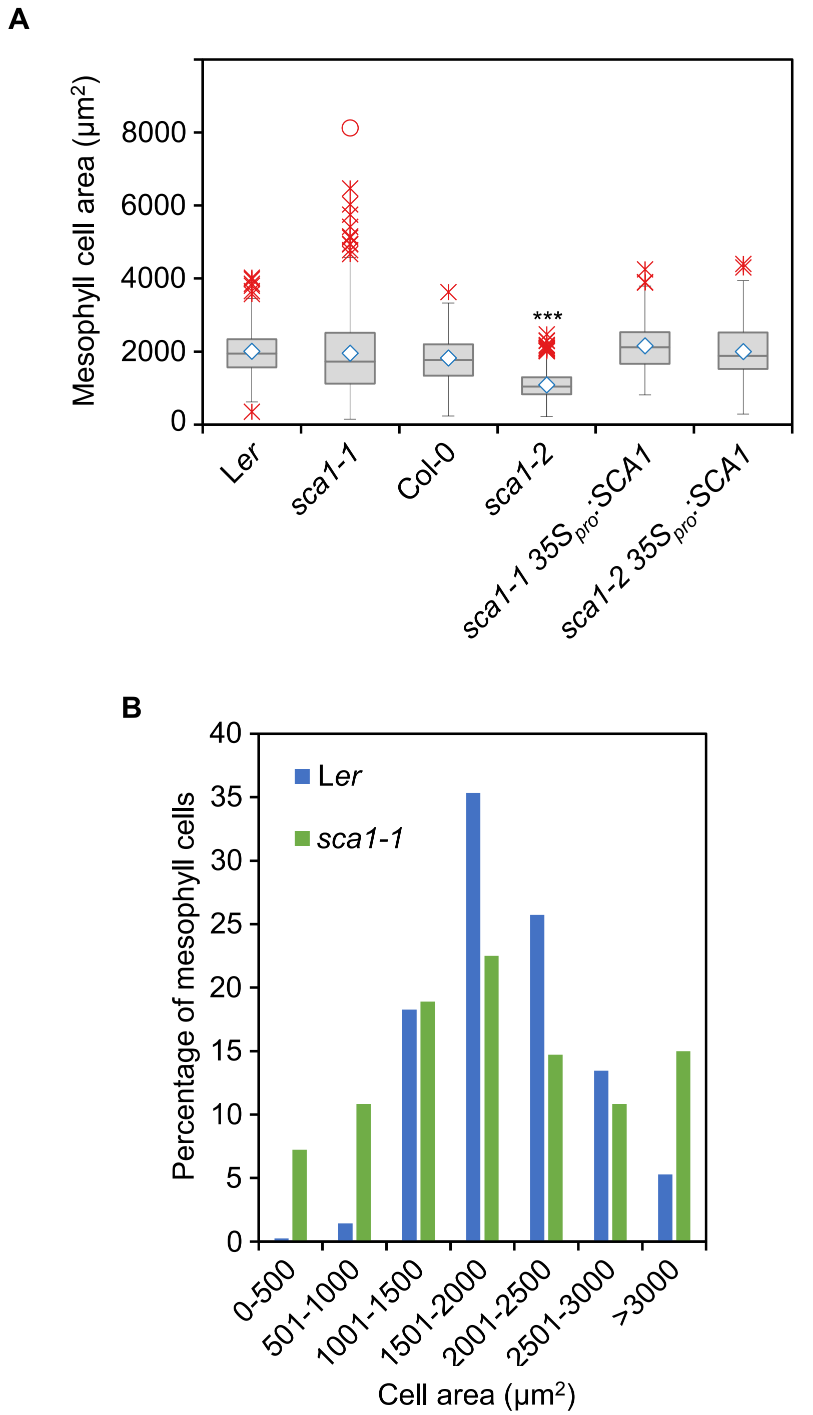

Supplementary Figure S3. Morphometric analysis of the palisade mesophyll cells of the *sca1* mutants. (A) Boxplot distribution of the palisade mesophyll cell area in first-node leaves from L*er*, *sca1-1*, Col-0, *sca1-2*, *sca1-1 35S$_{pro}$:SCA1* and *sca1-2 35S$_{pro}$:SCA1*. Boxes are delimited by the first (Q1, lower hinge) and third (Q3, upper hinge) quartiles. Whiskers represent Q1 - 1.5 IQ (lower) and Q3 + 1.5 IQ (upper), where IQ = Q3 - Q1. ◇: Mean. —: Median. O: Extreme maximum outlier (> [Q3 + 3·IQ]). ✱: Maximum or minimum outlier. (B) Distribution of palisade mesophyll cell area in first-node leaves from L*er* and *sca1-1*. More than 350 cells were analyzed from at least 10 different samples. Asterisks indicate a significant difference with the corresponding wild type in a Student's *t* test (****p*<0.001).
